# Noise patterns in visceral surgical procedures: Analysis of second-by-second dBA data of 599 procedures over the course of one year

**DOI:** 10.1038/s41598-020-59816-4

**Published:** 2020-02-20

**Authors:** C. T. Baltin, H. Wilhelm, M. Wittland, A. H. Hoelscher, D. Stippel, A. Astvatsatourov

**Affiliations:** 1 Department of Orthopaedics and Trauma Surgery, University Hospital of Cologne, Cologne, Germany; 2 Faculty of Management and Economics, Witten/Herdecke University, Witten, Germany; 3 Department of Nursing and Health Care, Faculty V, University of Applied Sciences and Arts, Hannover, Germany; 4 Contilia Centre for Diseases of the Oesophagus, Elisabeth Hospital Essen, Essen, Germany; 5 Department of General, Visceral and Cancer Surgery, University Hospital of Cologne, Cologne, Germany; 6 Clinical Trials Centre Cologne, University Hospital of Cologne, Cologne, Germany

**Keywords:** Preventive medicine, Occupational health, Risk factors

## Abstract

The objective of this study is to analyze noise patterns during 599 visceral surgical procedures. Considering work-safety regulations, we will identify immanent noise patterns during major visceral surgeries. Increased levels of noise are known to have negative health impacts. Based on a very fine-grained data collection over a year, this study will introduce a new procedure for visual representation of intra-surgery noise progression and pave new paths for future research on noise reduction in visceral surgery. Digital decibel sound-level meters were used to record the total noise in three operating theatres in one-second cycles over a year. These data were matched to archival data on surgery characteristics. Because surgeries inherently vary in length, we developed a new procedure to normalize surgery times to run cross-surgery comparisons. Based on this procedure, dBA values were adjusted to each normalized time point. Noise-level patterns are presented for surgeries contingent on important surgery characteristics: 16 different surgery types, operation method, day/night time point and operation complexity (complexity levels 1–3). This serves to cover a wide spectrum of day-to-day surgeries. The noise patterns reveal significant sound level differences of about 1 dBA, with the most-common noise level being spread between 55 and 60 dBA. This indicates a sound situation in many of the surgeries studied likely to cause stress in patients and staff. Absolute and relative risks of meeting or exceeding 60 dBA differ considerably across operation types. In conclusion, the study reveals that maximum noise levels of 55 dBA are frequently exceeded during visceral surgical procedures. Especially complex surgeries show, on average, a higher noise exposure. Our findings warrant active noise management for visceral surgery to reduce potential negative impacts of noise on surgical performance and outcome.

## Introduction

Surgeons, nurses and patients—during some procedures—are exposed to high noise levels. Noise is defined by work-safety regulations as a sound that may have a negative impact on health (e.g. impairment of hearing ability). Previous research demonstrates that the use of surgical instruments may result in significant noise exposure up to a level of 131 dBA for both staff and patient^1–3^. Existing research also shows that exposure to noise can increase an operating surgeon’s blood-based cortisol level, result in irreversible hearing loss or cause cardiovascular diseases^4–7^. The negative impact of noise is not limited to direct effects on staff and patient health but also affects the patient indirectly by lowering the quality of the surgeon’s work. Specifically, research demonstrates that intraoperative noise, among other factors, impairs the mental concentration of the operating team, eventually resulting in postoperative complications (e.g. infections)^8–11^.

While research on noise in the operating theatre is a mature research area, existing literature continues to lack comprehensive empirical evidence of noise pollution during visceral surgical procedures. This shortcoming in literature is not surprising, considering that visceral surgical procedures—which usually do not use noisy surgical instruments—seem less prone to extreme noise events. Consistent with this assumption, the plethora of noise studies in the surgical context focuses on orthopaedic surgery^2,3,11^. This assumption, however, may require revision. Initial evidence on noise exposure during different surgical procedures at the Johns Hopkins Hospital (Unites States of America) demonstrates that the noise exposure in neurosurgical, urologic and gastro-intestinal surgeries ranges between 62 and 65 dBA. These noise levels in visceral surgery are problematic, as they may harm patient and staff health^12,13^. They may also violate work-safety regulations^14^. Yet, without an encompassing investigation of processual indicators of noise in visceral surgical procedures, we lack evidence that could provide a foundation for engaging with this potential problem (e.g. by guiding noise-aware design of instruments for visceral surgery).

To address this shortcoming in the literature, and considering the substantive negative consequences of noise in the surgery theatre, we conducted an encompassing investigation into the noise levels of a broad spectrum of visceral surgical procedures covering multiple iterations of each procedure. Because we collected fine-grained data on a large number of visceral surgeries, our study addresses a second, fundamental shortcoming in the larger literature on noise in the operating theatre. Specifically, the majority of evidence—irrespective of the surgery type covered—is limited to non-processual descriptive indicators (e.g. Mean Noise Level, Standard Deviation of Noise, Maximum Noise, Minimum Noise)^2,3,15^. Processual indicators, that is, descriptive data capturing the development of noise patterns during procedures, are generally rare. The limited evidence covers only single surgical operations in the volume progression^16,17^. Because we lack comprehensive processual descriptive data, our understanding of noise patterns during surgeries remains extremely partial. Specifically, we have no evidence of how noise patterns typically unfold during different types of surgeries.

To address this methodical issue—and provide a more-complete understanding of noise patterns during surgeries—we introduce a new visual representation and comparison of noise-progression patterns for different types and durations of surgeries. This procedure enables researchers to reveal differences in noise exposure in the course of time of various surgeries. Our findings based on this procedure pave new paths for future research on noise patterns. In particular, they may inspire new research on the subjective experience of noise during surgeries.

Finally, to improve our understanding of surgery-level correlates of noise, we identify factors associated with the risk of noise events exceeding common policy—and evidence—based threshold levels (i.e. 60 dBA, 65 dBA and 70 dBA). Overall, our study provides new evidence on noise patterns in visceral surgery, new methods to describe noise patterns and identifies previously unstudied correlates of noise in visceral surgery.

## Method

Our study covers an observation period of 12 months—that is, from 1 April 2015 (SPSS time stamp 13647259440.0) to 4 March 2016 (SPSS time stamp 13676463960.0). Overall—during the time frame of our study—there were n = 1,100 surgeries included from three identically designed visceral operating theatres in a large German university hospital. According to architects and engineers of the facilities, the surgery theatres were designed for a reverberation coefficient ≤ 0.6s ± 20%.

**Figure 1:**
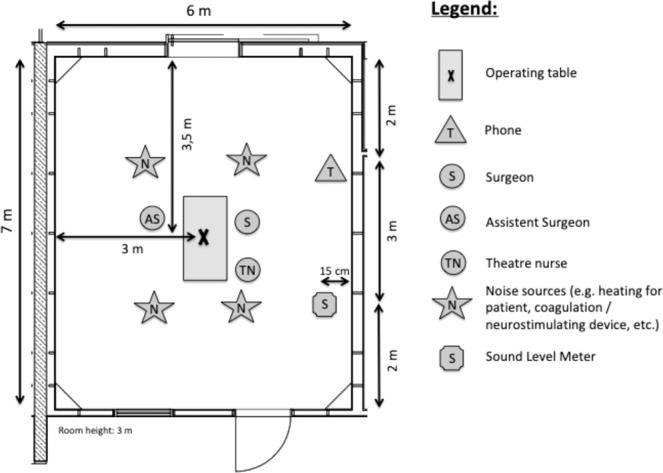
Layout of operating room including surgical team, material and the sound-level meter.

In each operating theatre, we measured the noise level exactly to the second using digital sound-level meters equipped with a recording function (PCE-322A, PCE Instruments, PCE Deutschland GmbH, Meschede). These decibel meters recorded the noise level in the “A” frequency-weighted setting (in dBA), which represents the standard mode for measurements in the occupational health and safety literature^18,19^. This mode is appropriate for the purpose of this study (i.e. to capture patterns of noise in visceral surgery) because it approximates the human auditory sensation using an integrated filter. In each of the three identically designed surgery theatres, the sound-level meter was mounted on an angle that was fixed to the wall. The sound-level meter was 15 cm away from the wall at a height of 1.3 metres, the height of the patient ear. We used this position to obtain the overall noise level in the theatre (evidence suggesting that a central position—as an alternative to our standard measurement position—is unlikely to result in substantially different results is available from the authors upon request). Figure 1 depicts the positioning of the sound-level meter in relation to the individuals and material in the room.

To cover a broad spectrum of visceral surgeries, we included both less-complex surgeries (e.g. port implantation) and very-complex surgeries (e.g. esophagectomy with gastric interposition or kidney transplantation) in the investigation. There were no a-priori exclusion criteria based on surgery types; even emergency procedures (e.g. splenectomies) were included. We did this to cast a wide net on different surgeries. The noise level and noise pattern across time results from various sources within the operating theatre during a surgery (e.g. surgical instruments, aspirator, telephone calls, alarms, conversations of the operating personnel, background noise, etc.). This encompassing measurement is consistent with the goal of our study to capture the overall noise during visceral surgery. Furthermore, given our goal to collect a large volume of many different visceral surgeries, resource limitations prohibited in-detail ethnographic observations of each surgery that would allow identification and singling-out of specific noise sources in our data. However, we collected additional data that provide tentative evidence on the typical sources of noise across the intra-surgery noise patterns in this discipline. Raw data from the sound-level meters were exported to a laptop every two days. This interval resulted from the internal storage capacity of the sound-level meters; it also allowed us to identify and resolve sound-level meter outages in a timely manner. Sound-level meter data were matched to archival surgery data using information on the surgery theatre and the time frame of the surgery. This allowed us to match additional information on the surgery (e.g. type of surgery) to dBA data.

We collected second-based noise data of n = 1,100 visceral surgeries (approx. 4.8 GB of raw data) during this time frame. Following data cleaning, reassessment and editing, a final sample of 599 surgeries with a total of 5,801,254 exact-to-the-second observations in dBA (approx. 2.5 GB of raw data) were included for further analysis in the study. There are technical as well as methodological reasons for the discrepancy between the number of surgeries and the number of observations covered by this study. Technically, the loss of observations in this long-term study partially resulted from occasional sound-level meter outages. The most common reason for these outages was technical failure caused by cleaning staff applying harsh detergents to the sound-level meters—despite several reminders to avoid this practice—as part of the day-to-day basic cleaning of the surgery theatres. Furthermore, some observation days lacked complete coverage with dBA data. Our investigations into how these gaps occurred uncovered that cleaners occasionally and unintentionally pulled the power cord from the sound-level meter when conducting last-minute cleaning before or after a surgery. We excluded corresponding surgeries from subsequent analysis to avoid biased results. Methodologically, to ensure comparability of dBA levels within a given procedure, we needed to ensure that the practices executed in a procedure are similar across iterations. However, very rare or one-of-a-kind procedures (e.g. specific wound revisions) lack such comparability. Therefore, we excluded such procedures.

In the end, we included 16 different procedures in our analysis. This serves to ensure valid comparisons across different iterations of the same procedure. Fortunately, the appearance of accidental artefacts with unusually high or low values during the measurement was very rare and could be automatically excluded from the final analysis. The data analysis was conducted via SPSS (Version 25) and R (Version 3.4.1).

All procedures performed in studies involving human participants were in accordance with the ethical standards of the institutional and national research committee and with the 1964 Helsinki declaration and its later amendments or comparable ethical standards. The ethics committee of the University Hospital of Cologne, Germany, approved all experimental protocols of this study. Informed consent was obtained from all individual participants included in the study.

### Analysis and results

To ensure comparability across surgery types, we included 16 types of operations in our sample. The number of records and main descriptive parameters for these 16 sets are presented in Table 1. Here, N_OP_ is the number of carried-out operations of a certain type, N_rec_ the number of collected sound levels, µ_DBA_ and SD_DBA_ the mean and standard deviation of the noise level in dBA over all operations of certain type, Q1, Median and Q3 are the interquartile ranges of 25%, 50% and 75%, respectively.

**Table 1:** Overview of the examined operations.

	Operation type	N_OP_	N_rec_	μ_DBA_	SD _DBA_	SEM _DBA_	(Q1-2*IQR)*	Q1	Median	Q3	(Q3 + 2*IQR)*
1	thyroidectomy	62	529,768	58.7	4.44	0.081	44.7	55.5	57.6	60.9	71.1
2	venous access surgery	50	104,386	58.9	4.54	0.182	43.6	55.6	58.3	61.6	73.6
3	splenectomy	10	82,270	61.4	4.74	0.214	52.3	57.5	60.5	65.2	80.6
4	esophagectomy	115	2,128,754	59.8	4.28	0.041	45.5	56.7	58.9	62.3	73.5
5	fundoplication	14	109,874	59.4	3.49	0.179	48.6	57.0	58.8	61.2	69.6
6	colonic resection	34	317,127	59.4	4.12	0.105	45.8	56.4	58.9	61.7	72.3
7	enterostomy	41	295,892	58.8	4.02	0.108	45.4	55.8	58.0	61.0	71.4
8	appendectomy	34	163,294	59.2	3.90	0.146	47.5	56.6	58.3	61.0	70.0
9	rectum resection	14	207,554	61.0	5.01	0.134	41.0	57.0	60.0	65.0	81.0
10	perianal abscess	22	22,822	58.7	4.98	0.389	42.3	55.3	58.4	61.8	74.8
11	partial liver resection	37	329,917	60.0	4.76	0.104	44.5	56.7	59.3	62.8	75.0
12	inguinal hernia	20	113,541	58.3	3.52	0.173	50.4	55.8	57.6	60.0	68.4
13	gastrectomy	41	588,282	58.9	4.36	0.077	45.3	55.9	58.0	61.2	71.8
14	cholecystectomy	51	352,224	59.2	4.19	0.100	46.1	56.3	58.5	61.4	71.6
15	nephrectomy	31	228,211	59.0	3.77	0.124	46.6	56.1	58.3	61.2	71.4
16	renal transplantation	23	227,338	58.7	4.26	0.123	44.2	55.6	58.1	61.3	72.2
	total	599	5,801,254								

**Figure 2:**
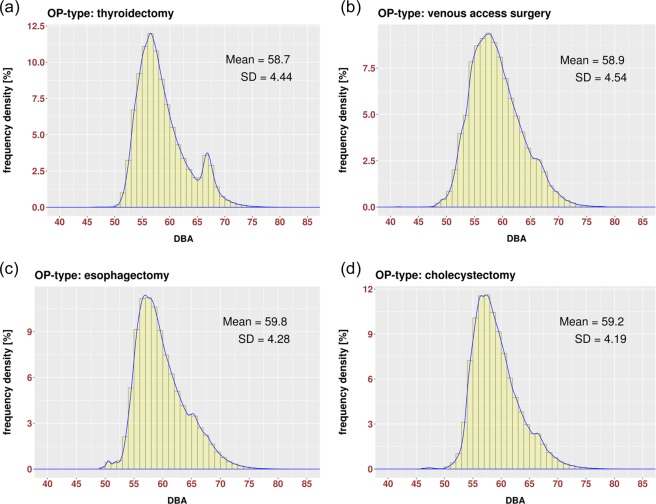
Noise-level distributions for operation types: (**a**) “thyroidectomy”, (**b**) “venous access surgery”, (**c**) “esophagectomy” and (**d**) “cholecystectomy”. These are the most-frequent operations. Each type contains 50 or more recorded operations (compare Table 1).

The noise-level distributions in dBA for the most-frequent operation types are presented in Fig. 2. Here, the y-axis represents the frequency density in percent. These distributions show that almost 100% of time the surgical team works in a loud environment (i.e. >50 dBA). About 40% of the time, they are working in an environment exhibiting a noise level higher than 60 dBA.

**Figure 3:**
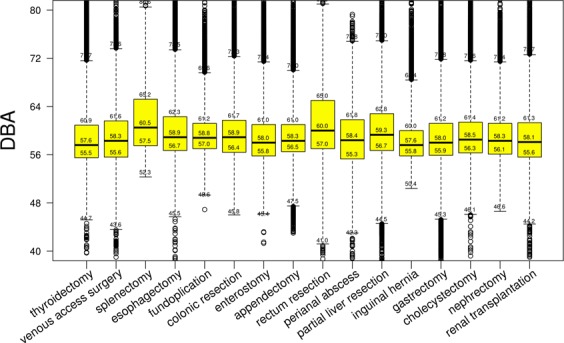
Boxplots for noise-level distributions of 16 operation types listed in Table 1.

The distributions presented in Fig. 2 have an asymmetric Gaussian form shifted in direction of the higher values and with the representative amplification around 65–70 dBA, which could be induced by specific noise factors, such as a coagulation device, increased conversations towards the end of a surgical procedure, etc. The noise-amplification effect is mostly expressed in distribution for thyroidectomy. In consequence of the asymmetric shapes, the noise distributions have different mean and median values. Both means and medians are listed in Table 1. Boxplots of the data collected in the selected 16 operation types (Table 1) are presented in Fig. 3.

Both Figs. 2 and 3 show that there are only a few isolated records (outliers) lower than 50 dBA and higher than 70 dBA. The number of sound records in regions lower than 50 dBA and higher than 70 dBA is negligible and the most usual noise level is around 55–60 dBA. The colored boxes in Fig. 2 represent the Q1–Q3 interquartile range (IQR). The lower whisker is defined as the *max(*Q1-2*∙IQR, minimum)* and marked as (Q1-2∙IQR)* in Table 1. The higher whisker is defined as the *min(*Q3 + 2*∙IQR, maximum)* and marked as (Q3 + 2∙IQR)* in Table 1. Significantly less than 1% of the records are left beyond the whiskers. Extreme values, which are lower than 40 dBA and higher than 80 dBA, were left behind the graphical presentation of boxplots in Fig. 2 to close up the median and IQR values. Here, the median and IQR values, listed in Table 1, are represented by numbers inside the boxplots.

Figure 3 shows that the highest decibel levels occur in splenectomy (60.5 dBA; 4.74 SD), rectum resection (60.0 dBA; 5.01 SD) and partial liver resection (59.3 dBA; 4.76 SD). The lowest decibel levels occur in inguinal hernia surgery (57.6 dBA; 3.52 SD), thyroid surgery (57.6 dBA; 4.44 SD) and gastrectomy (58.0 dBA; 4.36 SD).

**Figure 4:**
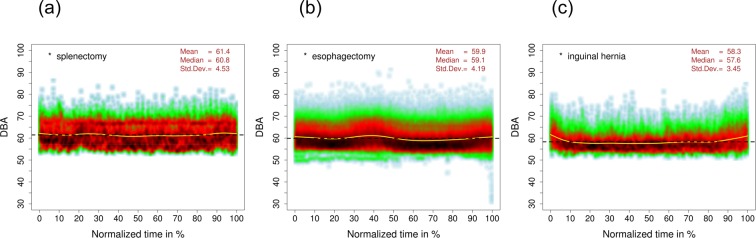
Smooth scatter plots: mean noise-level density distributions for three operation types: (**a**) splenectomy, (**b**) esophagectomy and (**c**) inguinal hernia, normalized on the operation time from 0% to 100%.

For each of the 599 surgical procedures, the data-collection time intervals (i.e. seconds) were transformed into the normalized time between 0% and 100% in 0.1% steps (i.e. each data set of individual operation was treated to consist of the 1,000 time stamps, so that each time stamp contains the mean value of the dBA records within this time stamp). Then the transformed data sets were combined in 16 sets, according to the corresponding operation type. Density distributions of the mean dBA records, treated in 1,000 time stamps, are shown in the smooth scatter plots of Fig. 4 for three operations types: a) splenectomy—with the highest noise level during the whole normalized operations period, b) esophagectomy—with the biggest noise level changes during the operationsand c) inguinal hernia—with the lowest noise level. The light-colored solid line in the middle of each smooth scatter plot of Fig. 4 represents the splines of the mean noise level, derived from each 10% of the normalized operation-time periods.

It is remarkable that splenectomy yields the highest noise level by far and an inguinal hernia surgery by far the lowest. Furthermore, it shows that for all surgeries the noise level rises at the beginning and at the end (always the first and the last 10–20% of the operating time).

For the 115 esophagectomies included in the investigation, a very distinct increase in noise after approximately 40% of the operating time was registered. This is ascribed to the repositioning action after termination of the gastrolysis as well as before the gastric interposition.

**Figure 5:**
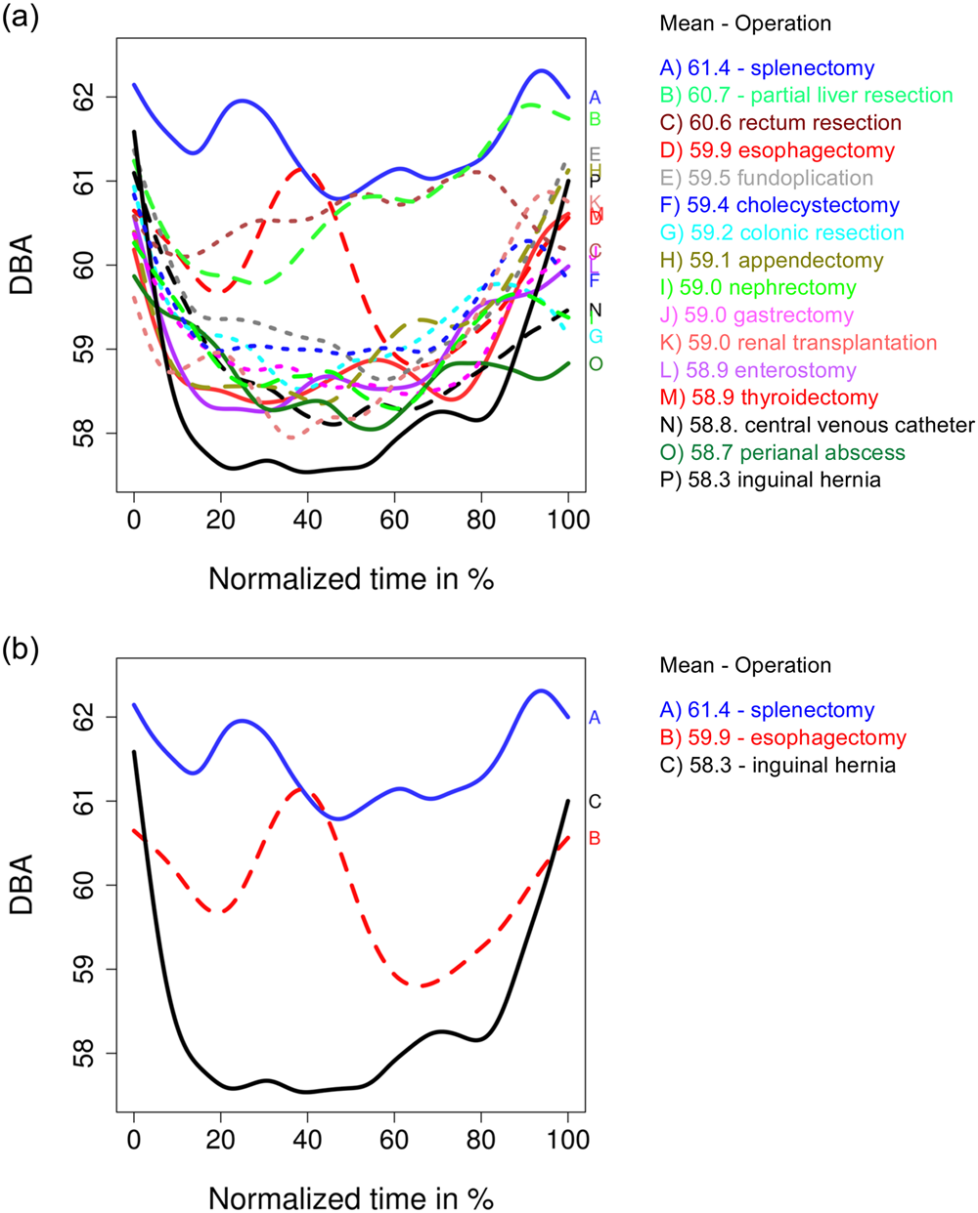
Splines of the mean noise level, derived from the distributions of Fig. 3: (**a**) for all 16 operation types; (**b**) selection of three operation types: splenectomy, esophagectomy and inguinal hernia, which have the highest, middle, but most changeable and the lowest mean noise level between 16 operation types.

The derived splines for all 16 operation types are shown in Fig. 5a. For an overview, the splines of the splenectomy, esophagectomy and inguinal hernia are selected in Fig. 5b. Overall, these figures demonstrate that there is a clear tendency of noisiness at the beginning and at the end of operations. The pattern of noisiness is evident–covering the preparation and clearance–the common noise level during the operation stays at very high level, oftentimes exceeding 58 dBA.

**Figure 6:**
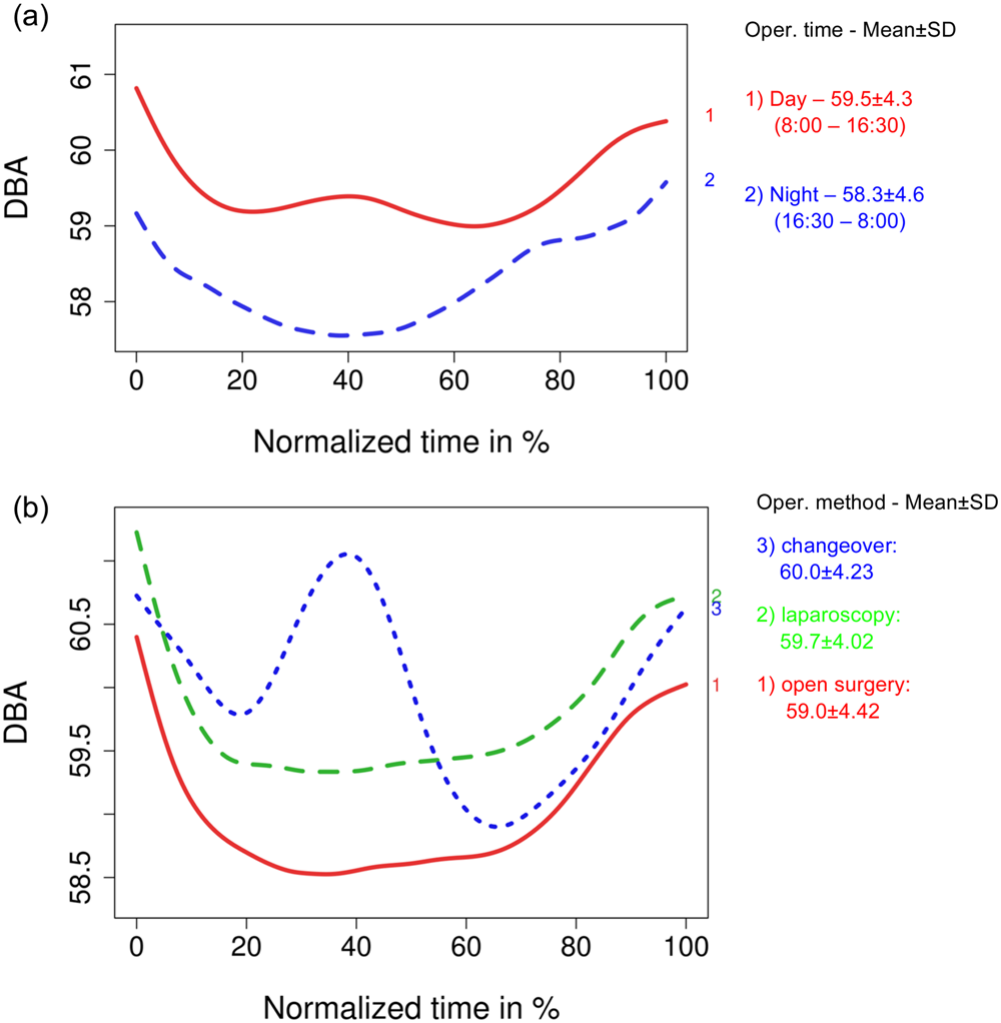
Splines of the noise-level distributions grouped by covariates: (**a**) operation time: day/night (**b**) operation method: open surgery/laparoscopy/conversion.

We also analyzed noise patterns in dependency on the covariates, such as operation time (defined as day/night; compare Fig. 6a) as well as operation method (open surgery vs. laparoscopic surgery including conversion from open surgery to laparoscopic surgery; compare Fig. 6b).

**Figure 7:**
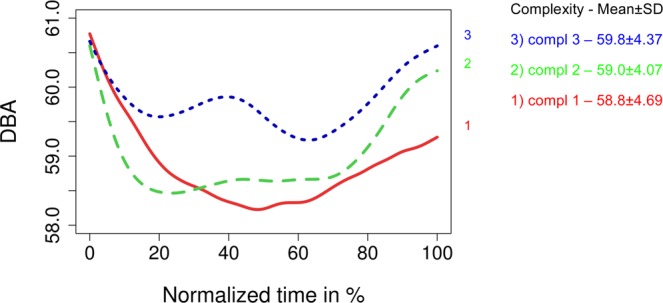
Splines of the mean noise-level distributions grouped by covariates: complexity of operation.

For additional analysis, we allocated surgery types to classes of more- orless-difficult surgeries. Specifically, less-complex surgeries (“complexity 1”) covered surgeries such as perianal abscess, implantation of venous access surgery, application of enterostomy or inguinal hernia. Medium-complex surgeries (“complexity 2”) covered procedures such as cholecystectomy, appendectomy or fundoplication. Finally, more-complex surgeries (“complexity 3”) covered procedures such as esophagectomy, splenectomy or kidney transplantation. Figure 7 presents the noise patterns contingent on the complexity of the surgery.

Comparison of Figs. 5–7 suggests that the overall noise level in visceral surgery usually exceeds 58 dBA. It also suggests substantial shifts in the noise levels within visceral surgeries as well as substantial difference in how these shifts occur depending on covariances, suggesting a broad spectrum of patterns of noise occurring in visceral surgery.

**Figure 8:**
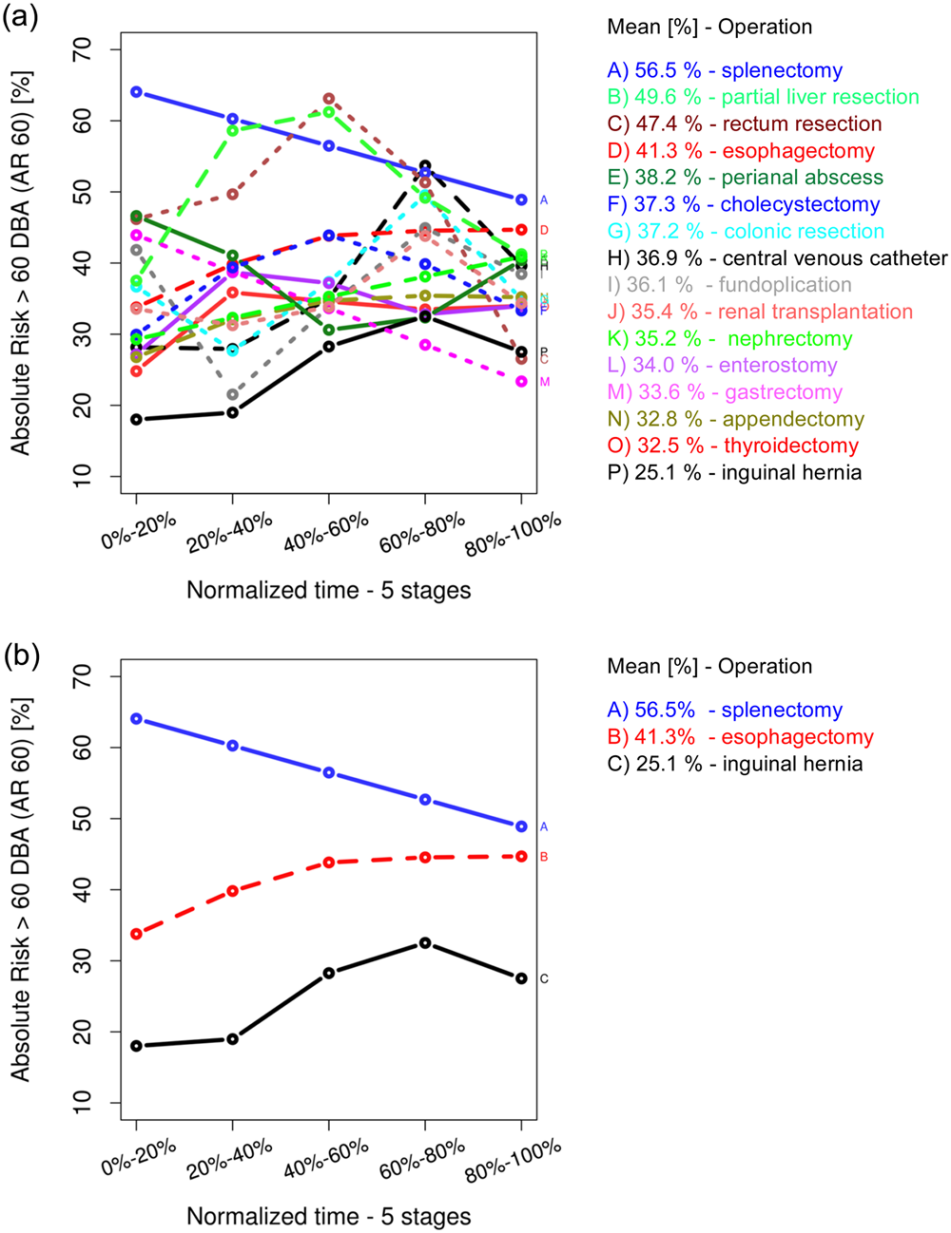
Absolute risk for noise level higher than 60 dBA: (**a**) for all 16 analyzed operation types, (**b**) focused on the absolute risk for splenectomy, esophagectomy and inguinal hernia.

Next, we investigated the risk of noise levels exceeding 60 dBA. The absolute risk of the 60 dBA or higher noise level was calculated as the rates ratio for five time intervals. Each interval contains continuously 20% of the normalized operation time. The rates ratio is the number of sound records higher or equal than 60 dBA versus all sound records in each interval: **AR**_**(≥60 dBA)**_** = N**_**(≥60 dBA)**_**/N**_**(all)**_. This analysis shows that the absolute risk for a noise event exceeding this level during one operation—e.g. for a splenectomy—was 56.5% on average. Interestingly, the risk for this event is significantly higher during the first half of the operation (i.e. between 56% to 65%), while the risk is significantly lower in the second half of the surgery (i.e. between 50% to 56%). For the partial removal of the liver, the rectum resection and the cholecystectomy the absolute risk of a noise event >60 dBA increases up until the first half of the operating time but then decreases until returning to the starting value (see Fig. 8).

The relative risks (RR) for each operation type were calculated as the ratios of the absolute risks (AR) for the corresponding operation type in respect to the AR of all other investigated operation types. For example, the RR_**(≥60 dBA)**_ for the operation type splenectomy is:

$$R{R}_{(\ge 60\,dBA)}^{\{splenectomy\}}=A{R}_{(\ge 60\,dBA)}^{\{splenectomy\}}/A{R}_{(\ge 60\,dBA)}^{\{all\,other\,operation\,types\}}.$$

**Figure 9:**
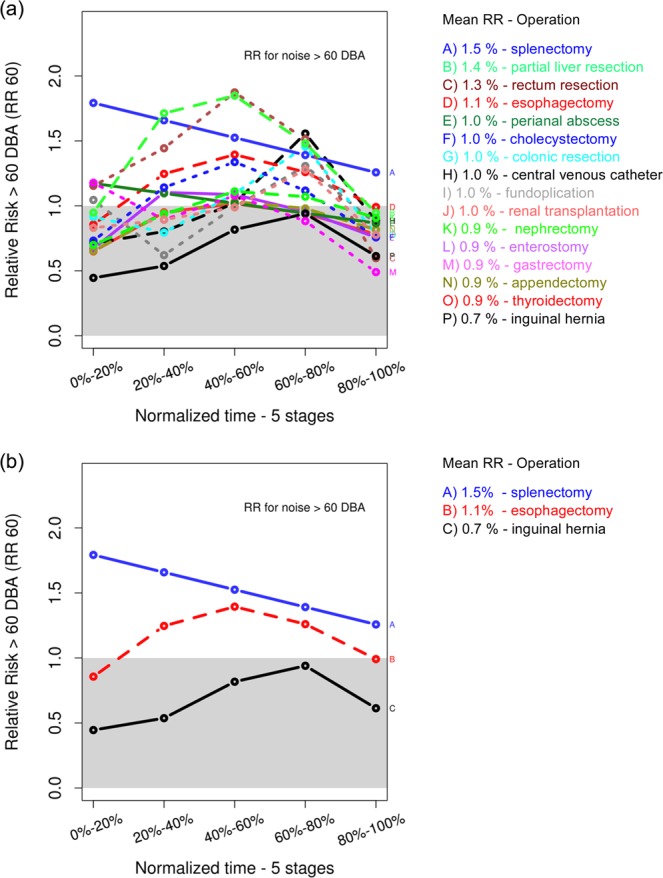
Relative risk for noise level higher than 60 dBA: (**a**) for all 16 analyzed operation types, (**b**) focused on the relative risk for splenectomy, esophagectomy and inguinal hernia.

The RR values (Fig. 9) show the “amplification” factor of one of the operation types in respect to the other operation types considered as the subsamples of the overall data.

## Discussion

The present study is one of the first to analyze an encompassing data set on noise patterns in visceral surgical procedures. These data were collected over the span of one year. Overall, 599 surgeries were extensively evaluated in this study. Prior to evaluating and interpreting these results, we first would like to highlight that the logarithmical unit decibel (0.1 Bel) is a dimensionless size of the sound-pressure level. In the present study, dBA spl (sound-pressure level) was measured in relation to the sound pressure 20 μPa. Thus, a reduction of 3 dBA equals a subjective noise reduction of 50%^20^. In consequence, while the differences in dBA documented in our above analysis may seem small at first sight, their practical difference in terms of noise is considerable.

Over the last years, noise exposure of patients and staff in operating theatres has received increased attention in both research and practice. This interest in noise management is reflected in the number of approaches for noise reduction in surgery theatres that have been proposed^16,21,22^. Such measures seem much needed, considering that current European and WHO guidelines for surgeon work recommend a maximum intra-surgery noise level of 55 dBA^13,23^. Current VDI guidelines (nr. 2058, issue 3) on the “Evaluation of noise at the workplace” point out that employees working in offices (or individuals doing similar light physical work) should not be exposed to noise levels exceeding 70 dBA. Finally, the norm DIN EN ISO 11690 Part 1 states target noise levels at industrial work sites (<80 dBA); office work (<55 dBA); and work requiring high levels of concentration (<45 dBA)^23^. Against the background of these norms, our results point out that visceral surgery—even when executed in technically up-to-date surgery environments—presents a problematic work setting because critical thresholds are commonly exceeded. For example, the smooth scatter plots printed in Fig. 4 document noise peaks of 80 to 90 dBA across a large number of surgeries. In other words, our data covering an observation time of one year and approximately 600 visceral surgical procedures show that noise levels of 58 dBA are exceeded on an a daily basis.

We would like to point out that there are some setting-specific noise generators that may affect our study. First, there is an ambient noise level—caused by air conditioning and other structural elements—that may vary between the operating theatres we studied and other surgery theatres. Due to the design of the surgery theatres we studied, this ambient noise level was identical for all three theatres where we collected our data. Second, the noise level in a university hospital setting, such as ours, could be elevated by the more-complex and sometimes life-threatening nature of surgeries executed in this setting. For example, this is the case for emergency procedures like splenectomies, which—as could be demonstrated—show highest decibel levels of all other surgeries, most likely due to an elevated blood loss and increased tension among the surgical team. Some surgeries also require more personnel, which could also elevate noise levels. Third, due to the academic mission of a university hospital, medical students or other staff usually attended surgeries. However, due to the positioning of the sound-level meter in the room, our results are unlikely to be primarily driven by student talk. Finally, events unrelated to the surgery—such as phone calls asking the surgeons to make urgent decisions on other patients—seem more common in university hospitals and may increase the noise level in the surgery theatres we studied.

Going beyond simple noise-level descriptive data, our study also provides novel insights into processual patterns of noise development in the operating theatre. Specifically, the splines analysis of different surgery types (Fig. 5) demonstrates that the noise patterns of many surgeries exhibit three typical phases. While the first and last phase exhibit a constant elevation of noise, the middle phase exhibits a lower noise level. The elevated noise level in the first phase may result from conversations between staff, increased movement by staff in the room and shifting of equipment. Elevated noise levels in the third phase likely result from increased communication between surgeons when executing the step-by-step wound closure or skin suture. Furthermore, arrangements for subsequent room change may result in additional noise. These results not only encourage future research on protocols and devices that limit noise in visceral surgery, the different phases we uncovered also provide guidance that interventions should focus on the first and final phase of surgery.

Considering possible strengths of our study, we would like to point out that our study offers a unique data set in terms of the number of surgeries and the detail of data collected for each surgery. Our results should generalize to a large number of visceral surgeries executed in modern surgery theatres exhibiting a reverberation coefficient ≤0.6s. Another strength of our data is that a Hawthorne Effect seems very unlikely. This is because of the extremely long data collection time (12 months) and the unobtrusive design and placement of the sound-level meter fostered quick habituation of the presence of sound-level recording. Additionally, at the beginning of our study, we communicated to all staff that the devices record noise levels only in dBA, but not communication content. Finally, we communicated that our study design does not allow matching of dBA to individual staff, further ruling out bias to social desirability.

Our study also has limitations that may provide a foundation for future research. Specifically, a weakness of the present study is that—due to the large number of observations and resource limitations—we could not collect matched intraoperative data on stress-induced hormonal cortisol variation or cardiovascular side effects (e.g. tachycardia or high blood pressure). Willich *et al*. showed that chronic noise exposure is associated with an increased risk for myocardial infarction^24^. Research has also demonstrated that increased cortisol levels resulting from noise are occasionally related to insulin resistance, stress ulcera or cardio vascular disease^24,25^. Against the backdrop of these studies and the results of our study—identifying a set of particularly noise-intense visceral surgeries—future research should focus on possible adverse health effects of noise for staff and patients in esophagectomy and partial liver resection. Furthermore—also due to the large number of observations and resource limitations—we could not employ a study design that distinguishes intra-surgery noise sources. For example, we relied on one sound-level meter per theatre. In consequence, our study only identifies inter-surgery correlates of noise. To address this limitation, future research should both rely on multiple sound-level meters in one surgery theatre as well as complementary qualitative data collection using non-participant observation.

Despite these limitations, our study provides a broad foundation of evidence suggesting that efforts to reduce noise in visceral surgery are much needed^16,26^. A multi-year, multi-centre study testing interventions to limit noise exposure in visceral surgeries and possible detrimental health effects seems timely.

## Conclusion

Our study—using a broad spectrum of surgeries, long data-collection time frame, and in consequence, a large amount of data—demonstrates that recommended maximum noise levels of 55 dBA are exceeded in visceral surgical theatres on a daily basis. While some contingencies lower the noise exposure (e.g. nocturnal noise exposure was on average 1 dBA lower than daytime noise exposure) and more-complex or emergency surgeries have per se a higher noise exposure on average of 1 dBA, active noise management is much needed in visceral surgery. Furthermore, our study points out new procedures to study noise in operating theatres on a more fine-grained procedural perspective.

Against the background of these results, we encourage future research seeking to improve protocols, techniques and instruments used in visceral surgery to limit noise exposure for patients and staff.
